# Neutralizing Nanobodies against Venoms from *Naja haje* Species Captured in North Africa

**DOI:** 10.3390/toxins16090393

**Published:** 2024-09-14

**Authors:** Hiba Mejri, Rym Mokrani, Ayoub Ksouri, Mabrouk Seddik, Nour Awad, Gabriel Ayme, Thouraya Chagour, Ahlem Mokrani, Charraf eddine Louchene, Imed Salhi, Rahma Ben Abderrazek, Rym Ben Khalifa, Zakaria Benlasfar, Pierre-Jean Corringer, Mohamed Hammadi, Selma Djilani, Pierre Lafaye, Balkiss Bouhaouala-Zahar

**Affiliations:** 1 Laboratory of Venoms and Theranostic Applications (LR20IPT01), Place Pasteur, BP704, Pasteur Institute of Tunis, Université Tunis el Manar, Tunis 1002, Tunisia; hiba.mejri@pasteur.utm.tn (H.M.); ayoub.ksouri@pasteur.utm.tn (A.K.); thouraya.chagour@pasteur.utm.tn (T.C.); rahma.benabderrazek@pasteur.tn (R.B.A.); benlasfarpas@gmail.com (Z.B.); rym.benkhalifa@pasteur.utm.tn (R.B.K.); 2 Antibody Engineering Platform, C2RT, Université de Paris Cité, CNRS UMR 3528, Institut Pasteur, 75015 Paris, France; gabriel.ayme@pasteur.fr (G.A.); pierre.lafaye@pasteur.fr (P.L.); 3 Research and Development Laboratory, Institut Pasteur Algérie, University of Algiers 1, Algiers 16000, Algeria; rmokrani@pasteur.dz (R.M.); amokrani@pasteur.dz (A.M.); celouchene@pasteur.dz (C.e.L.); sdjilani@pasteur.dz (S.D.); 4 Livestock and Wildlife Laboratory (LR16IRA04), Arid Lands Institute (I.R.A), University of Gabès, Medenine 4119, Tunisia; mabrouk.seddik@ira.rnrt.tn (M.S.); imed.salhi@ira.rnrt.tn (I.S.); mohamed.hammadi@ira.rnrt.tn (M.H.); 5 Channel Receptors Unit, Université de Paris Cité, CNRS UMR 3571, Institut Pasteur, 75015 Paris, France; nour.awad@pasteur.fr (N.A.); pierre-jean.corringer@pasteur.fr (P.-J.C.); 6 Faculté de Médecine de Tunis, Université Tunis el Manar, Tunis 1002, Tunisia

**Keywords:** *Naja haje*, cobra venom, LD50, neutralizing capacity, nanobody

## Abstract

Snakebite envenoming (SBE) remains a severely neglected public health issue, particularly affecting tropical and subtropical regions, with Africa experiencing an estimated 435,000 to 580,000 snakebites annually, leading to high morbidity and mortality rates, especially across Africa and Asia. Recognized as a Neglected Tropical Disease, SBE management is further complicated by the inadequate efficacy of current antivenom treatments. Of particular concern are cobras (*Naja* sp.), whose neurotoxins can induce rapid fatal respiratory paralysis. In this study, we investigate the potential of nanobodies as a promising next-generation of immunotherapeutics against cobra venoms. Through a dual strategy of the characterization of venom toxic fractions from cobras captured for the first time in Algeria and Tunisia biotopes, coupled with in vitro assays to evaluate their interactions with acetylcholine receptors, and subsequent immunization of dromedaries to produce specific nanobodies, we identified two lethal fractions, F5 and F6, from each venom, and selected five nanobodies with significant binding and neutralizing of 3DL50 (0.74 mg/kg). The combination of these nanobodies demonstrated a synergistic effect, reaching 100% neutralizing efficacy of 2DL50 lethal venom fraction (0.88 mg/kg) doses in mice. Additionally, our findings highlighted the complex mechanism of cobra venom action through the lethal synergism among its major toxins.

## 1. Introduction

Snakebite envenoming (SBE) remains a significant global health challenge, particularly in low- and middle-income countries. Recognized by the WHO as a neglected tropical disease in 2017, SBE globally results in 81,000 to 138,000 deaths annually, with a notable impact in Africa [1,2,3]. The WHO has highlighted the need for improved treatments and accessibility to antivenoms as part of its strategy to reduce the global burden of SBE. In Africa, over 40 species of Elapidae are documented [4], with approximately 22 belonging to the genus *Naja*. [5]. Specifically, in North Africa, the *Naja haje* sp. is prevalent. While the incidence rate of this species in North Africa is relatively low, it remains hypercritical, due to its potential risk for causing severe envenomations and fatalities. Regardless of the limited information available on *Naja haje* variability in North Africa, Broadley and Wüster have shown that, despite minor geographical and biotopic differences, the Moroccan populations of *Naja haje* could not be considered as a distinct subspecies [6]. In Tunisia, the *Naja haje* (Nht), characterized by its dark black and/or slight orange colors, is responsible for some fatal snakebites that occurred in the arid region. In Algeria, the *Naja haje* (Nha), distinguished by its dark yellow hue, contributes notably to local envenomation profiles.

Given the significance of *Naja haje* in North Africa, understanding the specific characteristics of its venom is essential. The venom from the *Naja haje* sp. is notably potent, characterized by rapid diffusing small neurotoxic peptides and cytotoxins (peptides of less than 10 kDa). The so-called neurotoxins, primarily Three-Finger Toxins (3FTxs), target postsynaptic nicotinic acetylcholine receptors (nAChRs), specifically [7]. Particularly, two subtypes are the primary targets: (i) the neuronal-type nicotinic receptors (α7-nAChRs), found in the central nervous system and responsible for cognitive functions and the modulation of neurotransmitter release, and (ii) the muscle-type nicotinic receptors ((α1)_2_β1δε-nAChRs), located at neuromuscular junctions and responsible for transmitting signals between nerve endings and skeletal muscles, to facilitate muscle contraction and mobility [8]. These neurotoxins have a strong ability to bind tightly to muscle-type nicotinic acetylcholine receptors (nAChRs), a key interaction detailed in ecent research [7]. By binding to these receptors, neurotoxins block the binding site for the neurotransmitter acetylcholine. Conventionally, acetylcholine binds to this site and helps to trigger muscle contractions. When neurotoxins block this interaction, the nerve signal transmission is disrupted, leading to paralysis of the skeletal muscles. This paralysis can become severe, extending to the main respiratory muscle, the diaphragm, which can lead to respiratory failure—a severe and life-threatening symptom of snakebite envenomation [9]. Considering the severe clinical manifestations, including potentially fatal paralysis, the need for more-effective antivenom therapies is very urgent.

However, current antivenom therapies, based on F(ab’)2 antibody fragments, primarily derived from equine- or ovine-animal producers, face several limitations, including variable effectiveness, a high risk of severe adverse reactions, logistical challenges in their production and distribution, and the additional challenge posed by their large molecular size [10,11,12,13]. This significant difference in molecular mass (and body diffusion) between the F(ab’)2 (90–110 kDa) and the venom toxins (6–7 kDa) is a major contributing factor to their limited efficacy, highlighting the need for more targeted and efficient therapeutic strategies for combating *Naja haje* SBE.

To overcome these challenges, a newly identified class of antibodies derived from camelids presents a promising approach to tackle the limitations associated with current available antivenoms. Nanobodies, or VHHs, are unique, due to their small size (~14 kDa), single-domain structure, and low immunogenicity [14,15], making them ideal candidates for antivenom development [16,17,18,19]. Derived from the heavy-chain-only antibodies found in camelids, these nanobodies are tailored to address the particular challenges posed by cobra venom. Their advantages extend beyond their biological and physicochemical properties. Their small size and robust nature facilitate easier and more cost-effective production in microbial and eukaryotic expression systems [16,20,21], storage, and transportation, addressing some of the key logistical challenges associated with current antivenom solutions. Moreover, their ability to neutralize venom toxins effectively, owing to excellent tissue permeability, positions them as a promising alternative in the quest to develop more efficient and safer antivenoms for SBE [22].

This study represents a first effort to investigate *Naja haje* in the North African context, specifically within Tunisia and Algeria, addressing a previously underexplored aspect of regional venom profiles. First, cobra specimens belonging to the subspecies *Naja haje haje* were captured from the two different biotopes. Dromedaries were immunized with the toxic fractions of both Nht (*Naja haje* from Tunisia) and Nha (*Naja haje* from Algeria) venoms, to generate specific nanobodies. These nanobodies were selected via phage-display library screenings, and subsequently tested against the venom’s toxic fractions. Initially, individual nanobodies demonstrated limited neutralizing capabilities against lethal doses of the toxic fractions. However, when combined in equimolar ratio, a set of five distinct nanobodies achieved 100% neutralization capacity, demonstrating a significantly improved neutralizing capacity, compared to the individually tested performance of each nanobody. This finding underscores the complex nature of snake venoms, which are composed of a diverse set of toxins.

## 2. Results

### 2.1. Purification of Naja haje Venom Fractions

**Figure 1:**
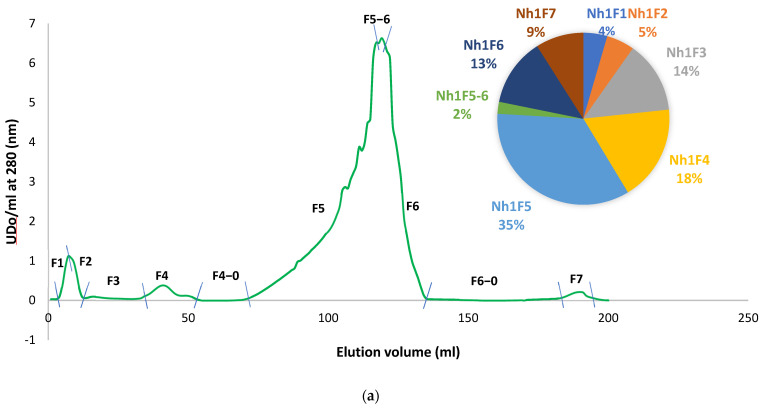

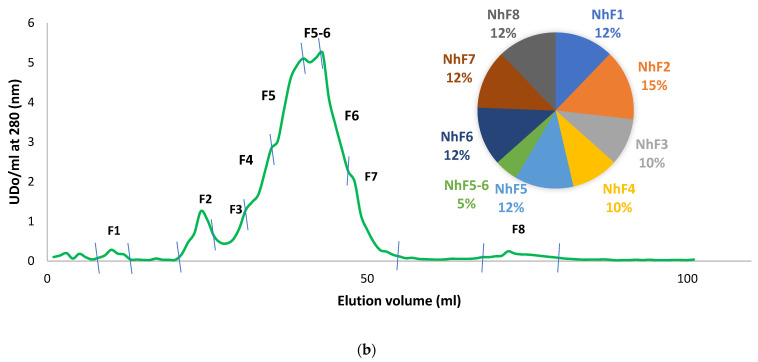
Size exclusion chromatography (SEC) profiles of *Naja haje* cobra venom extracts and estimation of main protein fractions. (**a**) Size exclusion chromatography of Nht venom with glacial acetic acid elution; (**b**) size exclusion chromatography of Nha venom with ammonium acetate elution.

Fractionation of Nht and/or Nha venoms was carried out using column chromatography with Sephadex G-50, employing either a glacial acetic or ammonium acetate buffer. This procedure resulted in the separation of the Nht and Nha venoms into seven distinct fractions, labeled as F1–F7 for Nht and F1–F8 for Nha, (Figure 1a,b). The percentages of each fraction are indicated in the corresponding figures.

### 2.2. Protein Biochip-Based Electrophoretic Profiles of Nht Venom Fractions

**Figure 2:**
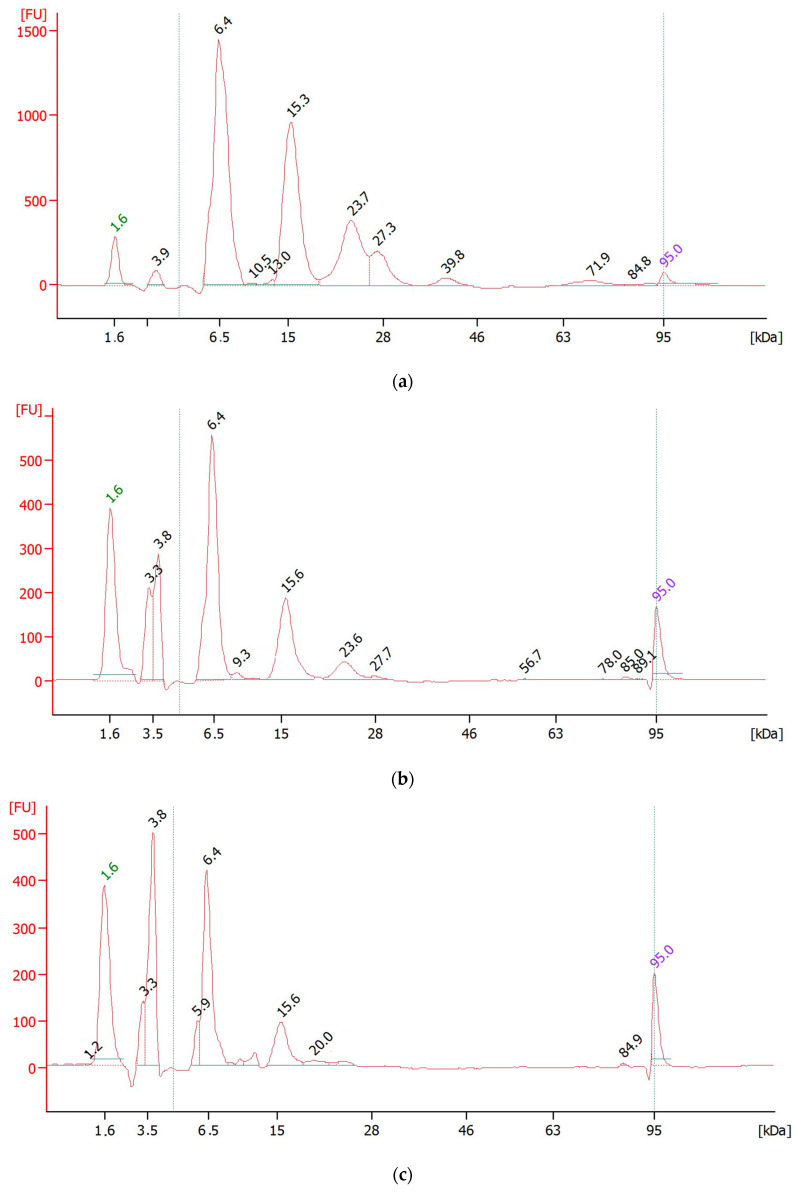
Analysis of Nht venom fractions via capillary electrophoresis, using an Agilent 2100 Bioanalyzer with a Protein 80 Kit: (**a**) Electropherogram of the Nht crude venom; (**b**) Electropherogram of NhtF5; (**c**) Electropherogram of NhtF6. The separation of proteins is shown within the molecular weight range of 1.6 to 95 kDa.

Nht crude venom and toxic fractions, obtained through size exclusion chromatography, were lyophilized and resuspended in sterile water to achieve a concentration of 0.5 µg/µL for electrophoresis. Including the whole venom pool, each sample (4 µL) was analyzed using the Agilent 2100 Bioanalyzer and the Protein 80 Kit (Figure 2).

Capillary electrophoresis of the Nht crude venom revealed a complex profile, with six distinct peaks at molecular weights of 6.4, 15.3, 23.7, 27.3, 39.8 and 71.9 kDa (Figure 2a). The analysis showed that the most abundant components were at 6.4 kDa (around 1500 FU (fluorescence unit), 48.5% of total venom), 15.3 kDa (1000 FU, 29.3%), and 23.7 kDa (400 FU, 13.8%), indicating a high concentration of small-molecular-weight proteins, which are typically associated with potent toxicity effect in Elapidae.

Upon fractionation, the profiles of toxic fractions NhtF5 and NhtF6 were notably simplified, containing fewer peaks. Fraction 5 (NhtF5) presented three major peaks, corresponding to 6.4 kDa (500 FU, 65.4%), 15.3 kDa (200 FU, 25.1%), and 23.7 kDa (50 FU, 6.5%) (Figure 2b). In contrast, Fraction 6 (NhtF6) showed only two peaks, at 6.4 kDa (400 FU, 64.4%) and 15.3 kDa (100 FU, 18.3%), with the absence of the 23.7 kDa component observed in Fraction 5 (Figure 2c). 

The peaks observed at 6.4 kDa, abundantly presented across all samples, predominantly correspond to neurotoxins, cardiotoxins, and other members of the three-finger toxin family according to Uniprot (P25675; P68418; P01457; P62394).

Capillary electrophoresis proved that the main toxic components, particularly neurotoxins and other small molecules from the three-finger toxin family, are highly concentrated in NhtF5 and NhtF6. These results justify the selection of these fractions as key targets for developing specific and effective nanobodies against elapid venom.

### 2.3. Median Lethal Dose Estimations

The median lethal dose (LD50) was estimated for the crude venom Nht (from Tunisia specimens) and Nha (from Algeria specimens) and for their respective toxic fractions, NhtF5, NhtF6, NhaF5 and NhaF6, using i.p. injections (500 µL/mouse, 20 g ± 2 g) mouse groups (Table 1). As a result, the LD50 of the Nht crude venom was found to be 6.64 µg/mouse (0.33 mg/kg), with observed symptoms including severe respiratory distress, paralysis, and convulsions, reflecting its potent toxic nature due to a complex mix, as confirmed in the capillary electrophoresis. The determined LD50 of the Nha crude venom was around 5.24 µg/mouse (0.26 mg/kg), showing similar symptoms, as observed with Nht. Significant toxic responses were observed in the Nh fractions. Specifically, NhtF5 induced behavioral abnormalities including disorientation, tremors, and muscle spasms, leading to an LD50 of 0.44 mg/kg. Similarly, NhtF6 caused flaccid paralysis starting in the hind limbs within one hour, progressing to the forelimbs by the fourth hour, with an LD50 of 0.74 mg/kg. Regarding NhaF5 and NhaF6, the LD50 values of 0.28 mg/kg and 0.25 mg/kg, respectively, along with the recorded symptoms, were largely similar to those observed with Nha crude venom. Detailed calculations and multiple trial results that contributed to these final values can be found in the Supplementary Data (Table S1).

### 2.4. Obtaining Specific Nanobodies by Phage-Display Library Screenings

Two dromedaries (females of 2 years old) were immunized against different toxic fractions of Nht and Nha cobra venoms, over a two-month period. One dromedary received the first two doses of the less toxic NhtF6 fraction, minimizing potential harm to the animal. Subsequently, the regimen was intensified with five doses of the more potent NhtF5 fraction. As a result, a significant increase in specific antibody titers was observed, starting from the 14th day and reaching a plateau by the 49th day, as determined through ELISA analysis (Supplemental Figure S1). The second dromedary followed a similar protocol with Nha venom fractions. 

**Figure 3:**
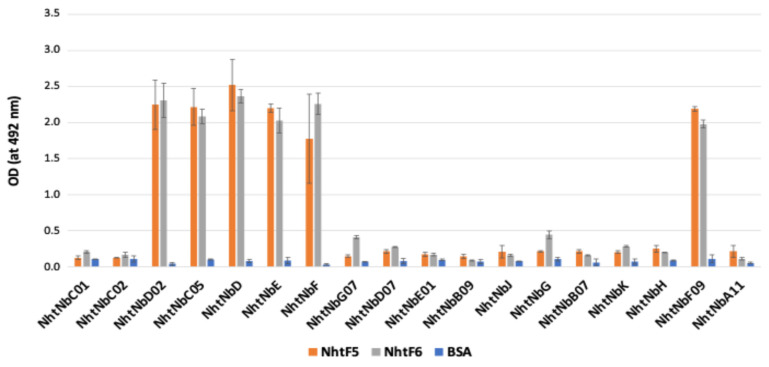
Binding capacity of the 18 selected nanobodies towards NhtF5/NhtF6 toxins determined by ELISA, in standardized conditions.

Upon dromedary immunizations, two libraries were constructed, L1 and L2, from cDNA encoding VHH domains isolated from peripheral lymphocytes of Nht-immunized dromedary and Nha-immunized dromedary, respectively. VHHs were selected by phage-display technology through three consecutive rounds of panning against both NhtF5 and NhtF6 for L1 and NhaF6 for L2, using progressively decreasing concentrations of antigen, different blocking agents and stringent washing conditions. At the end of the third round of panning, 400 individual clones from L1 and 86 from L2 were assayed by ELISA to test their binding capacity with NhtF5/NhtF6 and NhaF6, respectively. As a result, out of 400 individual clones tested, 90 clones were found positive towards binding NhtF5 and/or NhtF6 in phage-ELISA (54 for NhtF5 and 36 for NhtF6) compared to an irrelevant control (Supplemental Figure S2a), while out of 86 clones, only 2 clones were able to recognize and bind at equilibrium NhaF6 (Supplemental Figure S2b). The positive clones were sequenced using the Sanger method. Among the analyzed 90 DNA-selected sequences from L1, 12 clusters (identified as 1–12), with an additional 11 unique sequences (identified as A–K) were obtained. Each cluster corresponds to similar primary structures, with 2–3 point mutations in their amino acid sequences. The 11 unique sequences did not show any similarity to those in the established clusters. A total of 18 different Nbs were successfully produced (in pHEN6 or pET23a expression vector) and analyzed with SDS gel (Supplemental Figure S3). A standardized ELISA has shown that out of the 18 sequences produced, only 6 have demonstrated a significant binding capacity over the BSA control background (Figure 3). 

### 2.5. Toxic Fraction Binding to nAChRs Subtypes Expressed in HEK293 and Functional Characterization

**Figure 4:**
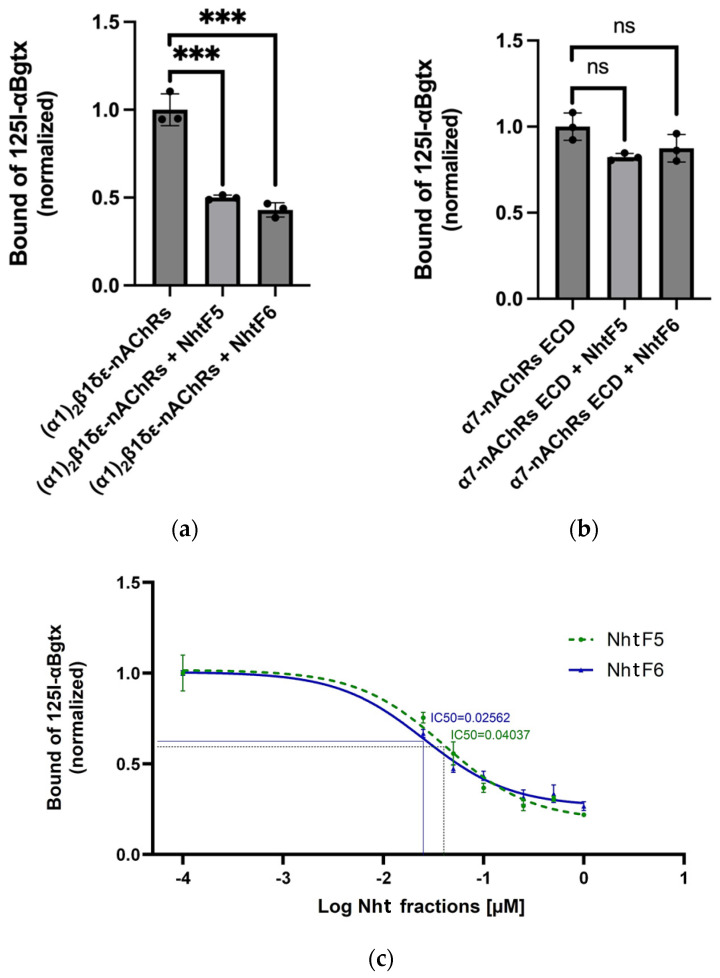
Specificity of NhtF5 and NhtF6 toxic fractions of cobra venom towards nAChRs subtypes. (**a**) Specificity of NhtF5 (100 nM) and NhtF6 (100 nM) towards (α1)_2_β1δε-nAChRs (muscle-type); (**b**) Specificity of NhtF5 (100 nM) and NhtF6 (100 nM) towards α7-nAChRs ECD (extracellular domain); (**c**) Dose–Response curve (IC50) of NhtF5 and NhtF6 towards (α1)_2_β1δε-nAChRs using I^125^α-Bungarotoxin (α-Bgtx). Statistical significance is denoted as ***: *p* < 0.001, ns: *p* > 0.05.

Competitive binding assays were conducted on HEK-293 cells expressing either the (α1)_2_β1δε -nAChRs (muscle-type) (Figure 4a) or α7-nAChRs ExtraCellular Domain (ECD) fused to the transmembrane domain of the 5HT_3_ receptor (neuronal-type) (Figure 4b) subtype. Following 1 h incubation of the transfected HEK-293 cells with the toxic fractions, I^125^ α-bungarotoxin (I^125^α-Bgtx) was added, to measure binding specificity. A high signal indicated no binding of the fractions, while a low signal suggested specificity in Toxin/Receptor complex interaction.

As a control, cells expressing nAChRs were incubated alone with I^125^ α-bungarotoxin (α-Bgtx), resulting in baseline counts per minute (cpm) of approximately 20,000 cpm for α7-nAChRs ECD and 10,000 cpm for (α1)_2_β1δε-nAChRs receptor subtypes. Notable modulation in the I^125^ α-Bgtx binding by both tested NhtF5 and NhtF6 was observed with the (α1)_2_β1δε-nAChRs receptor, showing the decreasing of the cpm to approximately 5000 for each fraction, indicating significant binding to this receptor subtype (Figure 4a). Conversely, no competition was detected for binding the α7-nAChRs ECD receptor with either fraction, suggesting no interaction with this subtype. This result clearly indicates a specific interaction of fractions NhtF5 and NhtF6 with the (α1)_2_β1δε-nAChRs receptor, but not with the α7-nAChRs ECD subtype (Figure 4b).

Dose–response relationships were quantified to assess the inhibitory effects of increased concentrations of toxic fractions NhtF5 and NhtF6 on the interaction between I^125^α-bungarotoxin (α-Bgtx) and the (α1)_2_β1δε-nAChRs receptor, using HEK-293 cells transfected with the nAChRs receptor. Concentrations of each toxic fraction tested were 0, 0.025, 0.050, 0.1, 0.25, 0.5, and 1 µM. Cells were pre-incubated with each concentration, prior to the addition of a fixed concentration of I^125^α-Bgtx (5nM). The data were logarithmically normalized to accurately demonstrate the concentration-dependent nature of the inhibition. The IC50 values, delineating the concentrations necessary to achieve a 50% reduction in α-BgTx binding, were calculated and estimated to be 40.37 nM (0.04037 µM) for NhtF5 and (25.62 nM (0.02562 µM) for NhtF6 (Figure 4c). These results were obtained from triplicate measurements.

### 2.6. In Vitro Analysis of Nanobody-Receptor Binding Affinity

**Figure 5:**
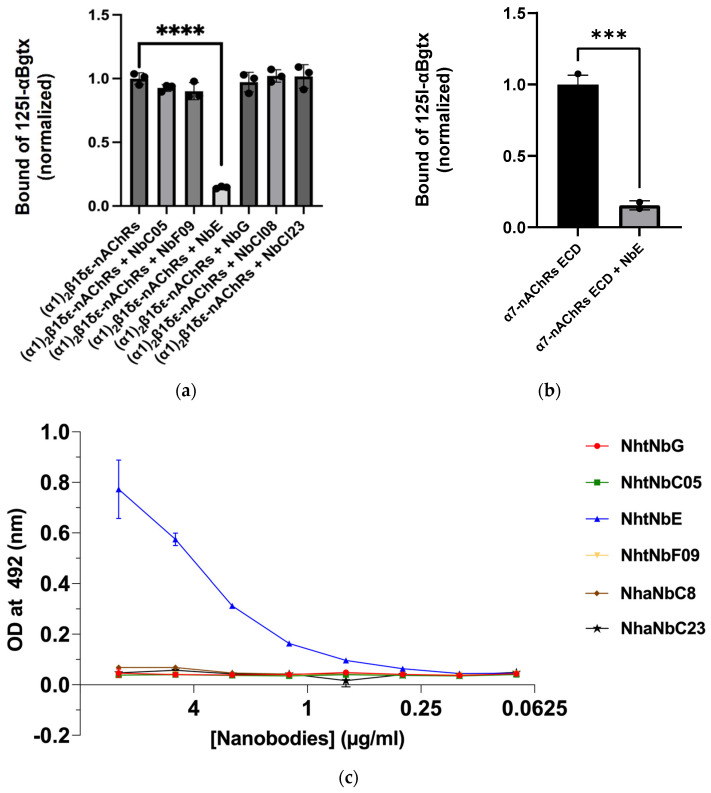
(**a**) Nanobody–(α1)_2_β1δε-nAChRs receptors’ binding affinity; (**b**) Nanobody-α7-nAChRs ECD receptors’ binding affinity; (**c**) ELISA evaluation of nanobody binding affinity to α-Bungarotoxin. ELISA plates were coated with α-bungarotoxin at a concentration of 1 µg/mL. Six nanobodies, initially at 10 µg/mL, were serially diluted and applied to assess binding affinity. Optical density was measured at 492 nm, to quantify the interaction between each nanobody and α-bungarotoxin. Statistical significance is denoted as ***: *p* < 0.001 and ****: *p* < 0.0001.

The inhibition efficacy of various nanobodies against (α1)_2_β1δε-nAChRs receptors is represented (Figure 5a), revealing the individual interaction of each nanobody as control. Amongst the different nanobodies, NbE emerges as a notable exception, achieving complete inhibition of the signal, while the rest exhibit minimal-to-no effect on α-Bgtx binding.

A focused analysis of NbE demonstrates its potential inhibition against the α7-nAChRs ECD receptor, with its consistent blocking activity (Figure 5b). This suggests that NbE has a unique and specific interaction with α-Bgtx that prevents its binding to both tested receptor subtypes. 

An ELISA assay was conducted to assess the interaction between various Nbs and immobilized α-Bgtx (Figure 5c). The assay revealed NbE binding to α-Bgtx. The binding affinities of the other Nbs to α-BgTx were comparatively lower, underscoring the unique binding properties of NbE.

### 2.7. Neutralizing Efficacy of Nanobodies

#### 2.7.1. Preliminary Finding

In this initial phase of our study, we evaluated the efficacy of the different nanobodies obtained (six nanobodies obtained from L1, which are NhtNbD02, NhtNbF, NhtNbF09, NhtNbD, NhtNbE and NhtNbC05, and two nanobodies obtained from L2, which are NhaNbCl08 and NhaNbCl23) (5.02 nmol/mouse) against 3LD_50_ of NhaF6 (0.74 mg/kg) (1:2 molar ratio NhaF6:Nb) in an in vivo setting using BALB/c mice. NhtNbG, which demonstrated very low binding capacity toward NhtF5 in standardized ELISA assays, was included as a negative control. The preliminary findings presented below in Table 2 outline the fact that out of the eight nanobodies, only five (NhtNbF09, NhtNbE, NhtNbC05, NhaNbCl08 and NhaNbCl23) demonstrate substantial neutralizing capacities of 3LD_50_. As expected, NhtNbG showed no neutralization effect.

#### 2.7.2. Analysis of Neutralization Efficacy

The neutralization capacity (NC) was assessed using the five nanobody candidates (Table 3). These nanobodies are distinct in their characteristics, including differences in CDR length, molecular weight (MW), and isoelectric point (pI). The detailed properties of these five nanobodies are provided in the Supplementary Data (Table S2). In this experiment, the selected nanobodies have been produced in eukaryotic HEK 293 cells to avoid the presence of endotoxins which might hamper the accuracy of the test. The purity of the nanobodies was checked using SDS-PAGE (Figure S4). The nanobodies were further purified with gel filtration chromatography (Cytiva-ÄKTA) and the nanobodies monomers were collected and used for NC studies (Figure S5).

Mice were administered with an intraperitoneal (i.p.) injection combining specific nanobodies with the 2LD_50_ of NhtF5 (0.88 mg/kg). This route ensures effective systemic absorption, necessary for observing symptoms related to diaphragm contractions and respiratory distress caused by cobra toxins. The nanobodies alone show substantial neutralizing capacities, especially at a 1–4 molar ratio, in favor of the considered Nb. The combination of the three nanobodies specific to the Tunisian cobra venom (NhtNbF09, NhtNbE, NhtNbC05) also show a moderate neutralizing capacity. Interestingly, the addition of the NhaCl08 and NhaCl23 specific to the Algerian cobra venom to the previous Nb-mixture shows a potent neutralizing activity. The neutralization capacity was improved with the enhanced amount of the nanobodies mixture used. Globally, 25% of the mice survived with the ratio 1:1, 50% with the ratio 1:2 and, finally, all the four mice survived (100%) at the ratio 1:4. The observed synergistic effect is strongly suggesting that the different nanobodies might neutralize structurally different venom toxin entities present in the NhtF5 fraction and/or through different venom toxin binding sites.

## 3. Discussion

Here, we describe a novel antivenom strategy achieved through the development of a group of five dromedary-derived nanobodies that can specifically target and completely neutralize the lethal fractions of *Naja haje* venoms of specimens captured from Tunisia and Algeria biotopes.

The goal was to develop nanobodies capable of recognizing and neutralizing both venoms, labeled Nht from Tunisia and Nha from Algeria, thereby ensuring effective cross-neutralization to cover sub-Saharan and North Africa regions.

This study included the evaluation of the toxicity of two crude *Naja haje* venoms via the intraperitoneal (i.p.) route in a murine model, utilizing the Spearman–Karber method. This i.p. approach has been previously validated as effective for testing various anti-toxin antibodies, providing reliable outcomes [23,24,25]. The i.p. route is particularly advantageous, as it simulates the rapid systemic distribution of venom following a snakebite, due to the highly vascularized mesothelial layer lining the peritoneal cavity, thus effectively mimicking the systemic effects of envenomation. The LD50 values obtained for the venoms, Nht from Tunisia (0.33 mg/kg) and Nha from Algeria (0.26 mg/kg), are consistent with previous findings for *Naja haje* venom from Egypt (0.25 mg/kg, intramuscular route) [26], and the Indian monocled cobra *Naja kaouthia* (0.22 mg/kg, intravenous route) [27], suggesting a high toxicity pattern within this species across different geographical regions. The toxicity of the *Naja haje* venoms from Tunisia and Algeria seems to be significantly higher compared to other *Naja* species evaluated using the i.p. injection route. For example, *Naja naja karachiensis* from Pakistan has an LD50 of 2.0 mg/kg [28] and *Naja naja atra* from China has an LD50 of 0.89 mg/kg [29].

Using size exclusion chromatography (SEC), we identified two major lethal fractions, F5 and F6. The study of LD50 for fractions F5 and F6 showed lower toxicity than the whole venom, suggesting that, following subsequent purification processes, the synergistic interactions among neurotoxins and fraction components are less important, thus reducing overall lethality. Our results are in accordance with previously published studies [30,31,32]. Moreover, in accordance with our expertise, the symptoms of neurotoxicity observed with cobra venom are distinct from those associated with scorpion neurotoxins. Cobra venom typically leads to paralysis, difficulty moving, muscle weakness, respiratory distress due to muscle paralysis, and convulsions. Additional symptoms include confusion, tremors, and behavioral difficulties, with some cases showing bending of the back correlated to diaphragm muscle contraction. Those symptomatologic observations are divergent from what is recorded with scorpion neurotoxins, which tend to cause malignant hyperthermia, myocarditis, and pulmonary edema. These effects are due to the venom’s low-molecular-weight proteins (neurotoxins), which primarily act on sodium (Na⁺) and potassium (K⁺) voltage-gated ion channels, affecting the electrical impulse conduction in most excitable tissues by altering ion permeability and action potential initiation. These differences highlight the distinct mechanisms of action between cobra and scorpion venoms, as further detailed in our recent work on BotI [19].

Capillary electrophoresis revealed that these cobra venom fractions F5 and F6 comprised two-to-three distinct MW protein families. Among these, the most abundant family, at approximately 6.4 kDa, was identified as three-finger toxins (3FTxs). According to UniProt (P25675; P68418; P01457; P62394), these toxins are known to include neurotoxins and cardiotoxins, which cause neuromuscular blockade and can lead to paralysis and respiratory failure [33].

Further investigation, including binding assays with nicotinic acetylcholine receptor (nAChR) subtypes, demonstrated the specificity of those two fractions to (α1)_2_β1δε-nAChRs (muscle-type). Those findings pointed out the importance of F5 and F6, making them key targets for developing potent neutralizing nanobodies against *Naja haje* venom’s.

To address the toxicity of those identified fractions, a total of 20 nanobodies were developed from phage-display screening of two VHH libraries and produced initially in the prokaryotic system (18 nanobodies from L1 and 2 nanobodies from L2). Among these, five nanobodies showed promising neutralization capacity. These promising nanobodies were selected and produced in HEK 293 eukaryotic cells to ensure proper folding and the absence of endotoxins, which are critical for their effectiveness in neutralization assays [34].

Analysis of nanobody–receptor binding affinity revealed that NbE, among the five nanobodies tested, exhibits unique properties. In the absence of the *Naja haje* toxic fractions, NbE showed an ability to bind to I^125^ α-bungarotoxin, resulting in a reduced signal. This observation suggests that NbE can interact with α-bungarotoxin, a toxin from the many-banded krait (*Bungarus multicinctus*), despite not being specifically developed against it. This unexpected dual recognition suggests potential broad-spectrum neutralization capabilities against diverse toxins sharing structural or functional similarities. The observed cross-reactivity may be attributed to structural motifs or epitopes shared between the targeted toxic fractions of *Naja haje* and α-Bgtx.

It is important to address the superior neutralizing effect of NhaNbCl23 compared to other antibodies in our study. This result suggests that NhaNbCl23 is binding a common or overlapped conserved epitope site to several toxins present in the F5 fraction, whereas the other partially neutralizing Nbs are most probably binding specific epitope sites divergent from NhaNbCl23’s. The non-detectable cross-interaction of NhaNbCl23 with α-bungarotoxin (of Bungarus venom origin) can be explained because it targets a toxin that does not share significant similarity with α-bungarotoxin.

Given the complex mixture of toxins present in *Naja haje*’s fractions/venom, it was necessary to use a cocktail of nanobodies to ensure complete and effective neutralization. A study by C. Venkatesan [35] explored the development of a cocktail antiserum based on rabbit monoclonal antibodies targeting specific venom proteins from *Naja naja*, demonstrating that a combination of antibodies—each targeting a different protein fraction—was necessary to achieve optimal protection in envenomated mice. Similarly, in our approach, while individual nanobodies showed promising results, it was the strategic combination of five distinct nanobodies that enabled us to reach 100% neutralization of the lethal fraction dose of *Naja haje* venom.

Other methods for developing broad-spectrum antivenoms include the innovative approach presented by Khalek [36], which utilized recombinantly produced toxins to select antibodies from a synthetic human antibody library. This method aimed to mimic nAChR binding to 3FTx-L (Three-Finger-Toxin Long-Chain), improving both affinity and efficacy, and offers substantial protection in vivo against lethal venom challenges of Asian and African elapid snakes. However, the complex production process, which involves screening billions of candidates and requires advanced facilities, makes it less feasible in resource-constrained environments. Hall et al. [37], has isolated nanobodies against α-cobratoxin (α-Cbtx), a single toxin from *Naja kaouthia*. They also prepared an homodimeric antibody with the nanobodies fused to the human IgG1 Fc region (VHH-Fc) for prolonged serum persistence and higher avidity. Despite promising results in mice, no further studies have been published since 2013. Recently, four nanobodies against the southeast Asian cobra *Naja atra* have been obtained. All nanobodies prolonged mouse survival and only one partially protected the mice from lethal dose [38]. Using a similar methodology, the N24 selected nanobody showed its effectiveness in neutralizing the most toxic peak from *Naja naja oxiana* venom from Iran [39].

Similarly to our study, Benard-Valle [40] recently explored the development of oligoclonal mixtures of cross-neutralizing nanobodies aimed at neutralizing the complex compositions found in coral snake venoms (*Micrurus fulvius* (Eastern coral snake, US) and *Micrurus diastema* (variable coral snake, Mexico)). Their study significantly advances the understanding and feasibility of using nanobodies to create broad-spectrum antivenoms.

In our current work, we have successfully developed five specific nanobodies against different toxins found in venoms collected from dark-black and black–yellow *Naja haje* cobras. The next step in our research will involve exploring the potential of these nanobodies to be engineered into a multi-target or broad-spectrum antivenom. This approach has already been developed by Wade et al. [18]. They engineered multivalent nanobody-based proteins with nanobodies incorporated into the Quad scaffold. These molecules displayed a neutralizing effect against long-alpha neurotoxins from both *N. kaouthia* and the forest cobra *N. melanoleuca*. A cohesive, multi-target molecule could potentially enhance the antivenom’s ability to simultaneously neutralize multiple toxins present in snake venom, thereby increasing the efficacy and breadth of the treatment.

Moreover, supplementary multifactorial orthogonal experiments are considered for ongoing study, using large batches of each of the purified Nbs. The stability and formulation development will be considered. This will allow the identification of the most efficient Nb combinations and the optimized and required administration doses, in mice and bigger animals. Various ratios of nanobodies versus venoms will be tested, along with the impact of different administration routes on the performance of venom-neutralizing capacities, and these will be assessed; the most effective parameters and combinations for developing antivenom treatments will be reported.

## 4. Conclusions

Our study presents the development of nanobodies targeting the toxic fraction *Naja haje*, which has demonstrated high affinity for the neuromuscular receptor nAChRs. The combined application of these nanobodies has shown to neutralize the target venom antigens with significantly enhanced efficacy, as assessed in an in vivo model at a molar ratio of 1:20 (NhtF5:Nanobodies). This innovation marks a substantial advancement in the production of targeted antivenoms, focusing on clinically significant venom components. The promising results demonstrate the potential of these nanobody candidates to serve as a foundation for a more effective and specific next-generation of antivenoms for SBE therapeutic use. Future investigations will aim to study the structure–function relationships and further optimize and expand the application of these nanobodies, potentially setting a new standard in antivenom development.

## 5. Materials and Methods

### 5.1. Animal Ethical and Well-Being Considerations

**Figure 6:**
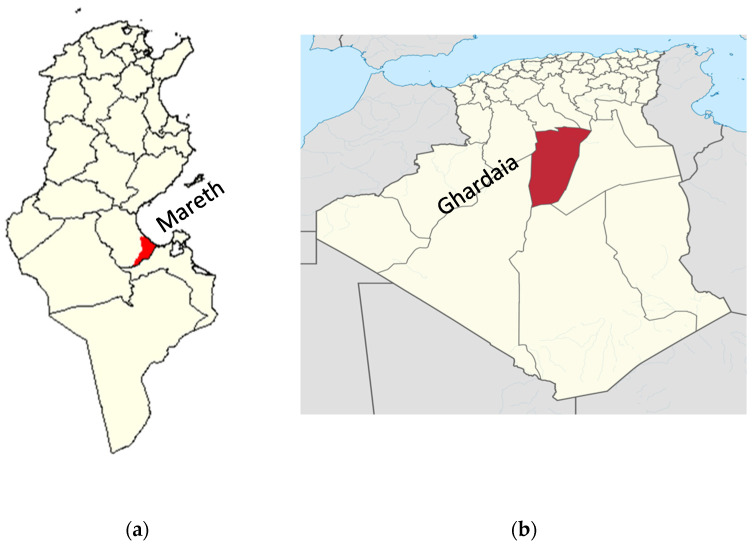
Map of the region from where Nh was collected. (**a**) Mareth, Tunisia; (**b**) Ghardaia, Algeria.

*Naja haje* (Nh) cobra specimens were captured from the south of Tunisia (Nht pool, Gabes, Tunisia) and from the center of Algeria (Nha pool, Ghardaia, Algeria) (Figure 6). Specimens were maintained in captivity, with appropriate feeding.

Two-year-old female dromedaries *Camelus dromedarius* (D1 (N°1925) and D2 (N°1917)), from the livestock of Institut des Regions Arides (IRA), Medenine, Tunisia, were immunized with Nht- and Nha-venom toxic fractions. All experiments on dromedaries have been approved by the local Biomedical Ethics Committee (ref. 2019/12/I/LR16IPT/V2) (CP47-19).

Swiss and Balb/C mice (8 weeks old, 20 (±2) g) used for toxicity and neutralizing assays, were provided by Institut Pasteur Tunis (Service des Unites Animalières) and Pasteur Institute Algeria (laboratoire des petits animaux). All animal testing experiments were carried out by experienced professionals. Testing protocols, established according to the WHO recommendations, have been early approved by the local Biomedical Ethics Committee (ref. 2019/12/I/LR16IPT/V2) (CP28-19).

The lab animals were randomly grouped and handled according to the institutional established procedures of acclimatation. To be precise, mice were housed in standard cages (EUROSTANDARD III Tecniplast^®^ 2190D) under controlled environmental conditions (22 ± 2 °C temperature, 65 ± 5% humidity, 55 decibel noise, and a 12 h light/dark cycle) with ad libitum access to standard rodent chow (CF3 granules, 8 mm Ø, EL BADR SA, Tunis, Tunisia) and water, ensuring optimal well-being. The study was designed in compliance with the 3R principles (Replacement, Reduction, and Refinement), to ensure ethical research and to minimize the impact on the animals’ well-being.

### 5.2. Snake Venom Milking and Characterization

#### 5.2.1. Snake Venom Milking

Nht and Nha snake venoms were manually collected into sterile tubes, by prompting them to bite through a parafilm surface. The collected pools of venoms are stored at −20 °C until used. Venom water extraction (*v*/3*v*) was performed, at +4 °C before the size-exclusion filtration step.

#### 5.2.2. Venom Size-Exclusion Chromatography

Nht and/or Nha venom pools were fractionated through size-exclusion chromatography using a Sephadex G50 (Sigma Aldrich, St. Louis, MO, USA 63178 États-Unis). The chromatographic column, measuring 2.5 cm in diameter and 100 cm in length, was pre-equilibrated with a 10% glacial acetic acid solution or ammonium acetate 100 mM, pH7. Each solubilized venom amount of approximately 50 uDO at 280 nm was uploaded onto the column. Venom fractions were collected at a consistent flow rate of 20 mL/h, 2 mL/fraction, and the optical density of each fraction was monitored at a wavelength of 280 nm. Each pool of peptides corresponding to distinct fractions was collected separately.

#### 5.2.3. Toxicity Assay and LD50 Determination of Nh Venom Fractions

Toxicity of crude venoms and toxic fractions (Nht and Nha) was evaluated, and the LD50 was determined on mice 20 g (±2 g), according to the ethically approved description of the protocol. First, the protein concentrations were measured using bicinchoninic acid reagent (BCA), as recently recommended [41,42,43]. The venom fraction dose was dissolved in an isotonic saline solution (0.9% sodium chloride) before injection. The LD50 was calculated according to the Spearman–Karber method [44,45]. Each toxicity testing was performed in triplicate, for reproducibility. Symptoms and/or deaths occurring within 24 h were recorded. 

#### 5.2.4. Capillary Electrophoresis and Biochip-Based Analysis of Nh Venom Fractions

Crude snake-venom-fraction protein composition was analyzed for protein integrity, using capillary electrophoresis (Agilent 2100 Bioanalyzer, Santa Clara, CA, USA), according to the manufacturer’s instructions. Briefly, 4 µL per sample was mixed with 2 µL sample buffer with DTT, for reduced conditions (Protein 80 Kit). Each sample was incubated at 95 °C for 5 min and further diluted with 84 µL water. A volume of 6 µL was applied to the biochip for analysis. (Agilent Protein 80 Kit Guide”, Agilent Technologies Manual, reference number G2938-90063. BIOTECHNIQUES VOL. 49, NO. 3) [46].

### 5.3. Dromedary Immunization Programs and Library Screenings

Well-established dromedary hyper-immunization programs (D1, N°1925 and D2, N°1917) were carried out separately, using two subcutaneous primo injections (250 µg and 350 µg at days 0 and 14, emulsioned in Freund’s complete adjuvant *v*/*v*) of less-toxic fractions (NhtF6 and NhaF6, respectively) followed by increased amounts of more-toxic fractions NhtF5 and NhaF5, respectively (150–400 µg/boost at days 21, 28, 35, 49 and 63, emulsioned in Freund’s incomplete adjuvant *v*/*v*). 

Before each injection, the dromedary’s blood was collected, and serum stored at −80 °C until use. The induced immune response was assessed by titrating serum Nht- and Nha-specific IgGs, using indirect ELISA assay. Nh-specific antibodies were revealed using polyclonal rabbit anti-dromedary HRP conjugate (Sigma Aldrich, St. Louis, USA).

Four days after the last boost, D1 and D2 bleeding was carried out, and a total volume of 200 mL was collected from the jugular vein for peripheral-blood mononuclear cell (PBMC) extraction using blood-density gradient centrifugation on Ficoll (2000 rpm, 20 min). The pellets of PBMCs were washed twice, before being stored at −80 °C. 

#### 5.3.1. Library Construction and VHH Cloning

Two VHH libraries (L1 from D1 and L2 from D2, respectively) were constructed using well-established procedures with slight modifications, using a set of specific primers [17,47,48]. Briefly, total RNA was extracted from about 10^8^ lymphocytes and 50–70 ug used as a template to synthesize 1st- and 2nd-strand cDNA by reverse transcription PCR, using Random hexamer and Superscript-II reverse transcriptase (Invitrogen, Illkirch, France). The obtained double-strand cDNA was used to amplify the VHH gene regions in a two-step nested PCR using different sets of primers, with SfiI or PstI and NotI adapters (Table S3). The VHH fragments (500 bp amplicons) were cloned using T4 ligase (New England Biolabs, Evry, France) into the pHEN6 for L1 and the pMECS for L2 phagemid vectors. The ligation mixes (different vector/insert ratio) were used to transform electrocompetent *E. coli* TG1 cells. Recombinant VHH clones were scratched and stored in −80 °C (in DMSO, 8% or Glycerol, 20%).

#### 5.3.2. Phage-Display Library Screening, Enrichments and Positive Clone Rescuing

Phage display was performed according to previously described protocols [17,49]. Briefly, phage-library L1 and L2 representative samples were used for *E. coli TG1* exponential growth (in LB, 1% glucose medium, Ampicillin 100 µg/mL, absorbance 0.5–0.6 uDO_600_) before being infected with M13K07 helper phage (New England Biolabs, Evry, France, 1/20 ratio). After 30 min of incubation at room temperature (RT) without stirring, the infected bacteria were incubated for 30 min at 37 °C, 150 rpm, Kanamycin 50 µg/mL. The phage-VHHs were induced in the presence of 1 mM IPTG overnight, at moderate temperatures for each library (30 °C and 28 °C for L1 and L2, respectively). Post induction, the bacterial cells were discarded by centrifugation (5000 rpm, 10 min) and Kanamycin-resistant recombinant phages were precipitated using polyethylene glycol (PEG_6000_)/NaCl and resuspended in phosphate-buffered saline (PBS, Dubelcco) for further panning. MaxiSorp Nunc Immuno-tubes (L1) or plates (L2) were used for coating the toxic venom fractions, with progressively decreasing concentrations of antigen at each round (from 10 µg/mL to 8 µg/mL) being employed. Three consecutive rounds of panning were performed against specific venom fractions (NhtF5 and NhtF6 for L1, and NhaF6 for L2), using different stringent conditions (2% skim milk, 3% BSA and Li-cor Odyssey diluted ¼ Blocking Buffers), from round to round, to enhance stringency and reduce non-specific binding. For the washing step, progressively higher Tween 20 concentrations (from 0.1 to 0.5%) of PBST were used, to remove non-specifically bound phages. Rescued phages were then eluted with 100 mM triethylamine (1 mL, TEA) and the pH was immediately neutralized with 2 M Tris-HCl (0.5 mL, pH 7.4), then used to infect exponentially growing *E. coli* TG1 cells. 

#### 5.3.3. Nanobody Selection Using Enzyme-Linked Immunosorbent Assays

At the end of panning, library enrichment for specific VHH-displaying phages or periplasmic extracts (PEs) was assessed, using ELISA-based assays. Briefly, individual colonies were randomly picked from the third round and O/N incubated with shaking at 37 °C in 96-deep well plates containing 2YT medium supplemented with ampicillin (100 µg/mL). The growing host cells were cultured (single PE-ELISA) or infected with M13KO7 helper phages and tested (single-phage ELISA). The growing host cells were then either cultured (single PE-ELISA) or infected with M13KO7 helper phages (single-phage ELISA). Both cultures were induced with 1 mM IPTG and grown overnight. The next day, the plates were centrifuged (30 min at 2500 rpm). For PE-ELISA, the cells were resuspended in cold TES buffer (20% sucrose, 30 mM Tris-HCl, 1 mM EDTA) and stirred at 4 °C for 30 min, then diluted 1–4 with TES buffer, and stirred again at 4 °C for 30 min. After centrifugation, the resulting PE extract was used for the ELISA. For phage ELISA, the supernatant was used directly for the ELISA. Wells of an ELISA plate were coated overnight at 4 °C with 100 µL each of NhtF5 and NhtF6, NhaF6 (1–5 µg/mL), or BSA (1 µg/mL) as a control, then washed with PBST (0.1%) and incubated with recombinant phages (⅕ dilution) or PE extract, from the selected colonies in PGT (PBST 0.1%, 10% gelatin) at 37 °C for 1 h. After the washing step, the plates were incubated with rabbit anti-His (Novagen, Darmstadt, Germany) or anti-M13 antibodies (Sino Biologicals, Eschborn, Germany). Antibody complex interactions were revealed using an anti-rabbit HRP-conjugate (Vector or Abcam), for 1 h at 37 °C. Positive clones were selected, after adding TMB substrate, based on high absorbance measured at 492 nm values compared to control absorbance (BSA, Sigma-Aldrich, St. Louis, USA), and stored at −80 °C in Glycerol 20%.

Furthermore, the binding capacity of the selected and purified nanobodies (BCA standardized assay (Merck, Lyon, France), obtained through Ni-NTA (Qiagen, Courtaboeuf, France) purification was assessed at a concentration (5 µg/mL in PGT). The evaluation of their affinities towards NhtF5, NhtF6, NhaF6 (1 μg/mL of each venom fraction in 100 μL PBS) were estimated using the well-established indirect ELISA assay, on MaxiSorp wells (NUNC, Thermo Fisher, Illkirch, France). Binding interactions were revealed using anti-Histidine antibody (1:2000 dilution, Sigma-Aldrich, St. Louis, USA) followed by anti-rabbit HRP-conjugated (1:1000 dilution). TMB substrate (100 µL) was used, and color was stopped by adding 3 N H_2_SO_4_, before absorbance measurements (450 nm), using a Thermo Scientific ELISA reader.

#### 5.3.4. Nanobody Sequence Analysis

The cDNA inserts corresponding to the positive clones were sequenced (Eurofin genomics services—Germany). Each nucleotide sequence was translated into its amino acid sequence using EMBOSS Transeq software (V6.6.0) (https://www.ebi.ac.uk/jdispatcher/st/emboss_transeq accessed on 11 May 2022), and the resulting data were aligned with Clustal Omega (V1.2.4) (https://www.ebi.ac.uk/jdispatcher/msa/clustalo accessed on 11 May 2022) and, thereafter, analyzed using the Protparam software (V3.0) (https://web.expasy.org/protparam/ accessed on 19 July 2024).

### 5.4. Nanobody Expression and Purification Using Prokaryotic System

#### 5.4.1. Expression in pHEN6 Vector

The VHH sequences from positive clones, rescued by L1 biopanning and expressed in pHEN6 phagemid, contain a C-terminal His6-tag facilitating the expression and the purification of corresponding nanobodies in the periplasm compartment. Briefly, nanobody expression was IPTG-induced (0.5 mM at 30 °C, O/N). Then, the pellet of cells was resuspended in a lysis buffer supplemented with cOmplete, Mini, EDTA-free, protease-inhibitor cocktail tablets (Sigma Aldrich, Taufkirchen, Germany). The nanobodies were released through a vigorous shaking at 4 °C and purified using cobalt HisPur beads (Thermo Scientific, Waltham, MA, USA) on an Econo-pack column (Biorad, Hercules, CA, USA) employing an increasing gradient of imidazole concentration (ranging from 10 mM to 500 mM imidazole, pH 8.0) (Sigma).

#### 5.4.2. Expression in pET23a Vector

To optimize production yields of low-expressed positive clones, VHH sequences were subcloned into the pET23a plasmid, using the restriction-free (RF)-cloning method and transformed into *E. coli* BL21 (DE3) cells. Hereby, the expression occurred in the cytoplasm compartment under IPTG induction, as described above (Section 5.4.1). Then, cells mixed with Benzonase (Biovision, Luton, UK) were treated using a cell disruptor (Constant systems LTD, Northants, UK) to facilitate the release of nanobodies.

#### 5.4.3. Expression in pMECS Vector

The VHH sequences from positive clones, rescued by L2 biopanning, were expressed in the pMECS phagemid, which is similar to pHEN6, despite containing in its C-terminal extremity two tags (HA- and His6- tags), as previously described [16,50]. Briefly, recombinant VHH-pMECS phagemids were used to transform *E. coli* WK6 electrocompetent cells. Expression was induced with 1 mM IPTG at 28 °C for at least 16 h. Periplasmic proteins were extracted by osmotic shock, purified via IMAC using Nickel–Nitrilotriacetic acid (Ni-NTA) Superflow columns (Qiagen, Courtaboeuf, France).

### 5.5. Nanobodies’ Expression and Purification Using Eukaryotic System

For the best-in-class positive colonies expressing nanobodies with Nh-neutralizing activities, subcloning in the eukaryotic system was performed. Briefly, the Maxi kit for endotoxin-free plasmid DNA extraction (Macherey-Nagel, Duren, Germany) was used. Both pHEN6- and pFUSE-derived vectors were digested with NotI/NcoI restriction enzymes [51]. Insert and vector were T4 DNA ligase-ligated (New England BioLabs, Ipswich, MA, USA) and used to transform *E. coli* XL1-Blue competent cells (2YT medium containing Zeocin). The recombinant plasmid was used to transfect Expi293F mammalian cells (ThermoFisher, Illkirch, France) using the ExpiFectamine 293 Transfection Kit (Fisher Scientific, Waltham, MA, USA), according to the manufacturer’s instructions. The nanobodies were purified using cobalt HisPur beads (Thermo Scientific, Waltham, MA, USA) on an Econo-pack column (Bio-rad), using specific concentrations of Imidazole. The nanobodies were analyzed using SDS-PAGE and purified with gel filtration chromatography (Cytiva-ÄKTA).

### 5.6. SDS-PAGE Analysis of Purified Nanobodies

The purity of the eluted nanobodies was estimated using sodium dodecyl sulfate-polyacrylamide gel electrophoresis (SDS PAGE) followed by Coomassie blue staining. Then, protein concentrations of dialyzed nanobodies (in PBS buffer) were estimated using a copper-based BCA test (Thermo Scientific). The fractions, containing 99% of pure nanobodies, were collected, pooled, and concentrated using a 3000 molecular-weight cutoff ultrafiltration unit from Millipore (Thermo Scientific).

### 5.7. Radioactive I^125^α-Bgtx Competition Assay

HEK293 cells were cultured in high-glucose Dulbecco’s Modified Eagle Medium supplemented with 10% fetal bovine serum (DMEM-FBS) and an antibiotic mixture of 1% penicillin/streptomycin incubated at 37 °C with 5% CO_2_.

Before transfection, HEK-293 cells were seeded (at a density of 7 × 10^4^ per 100 mm dish) in DMEM-FBS supplemented with 1% penicillin/streptomycin. When cells were approximately 70–80% confluent after 48–72 h, cells were transfected with 10 µg of DNA, coding for either the extracellular domain of the α7-nAChRs receptor (chimeric protein: extracellular α7 domain + intramembrane serotonin (5HT_3_) domain) or the (α1)_2_β1δε-nAChRs receptor (muscle-type) construct using JetPrime reagent (Polypus), according to the manufacturer’s protocol, and were incubated for an additional 48 h. After incubation, 5 dishes (size 100 mm) at 10 × 10^6^ cells per dish were washed with PBS, detached and resuspended to individual cells in 10 mL of binding buffer (10 mM HEPES, 2.5 mM CaCl_2_, 2.5 mM MgCl_2_, 82.5 mM NaCl, pH 7.2) mixed with protease inhibitor EDTA-free (Sigma-Aldrich). For each sample, 150 µL of the cell resuspension was pipetted into glass vials in triplicate. A total of 100 nM of each toxic fraction to be tested was added into the control and ACh (1 mM) samples, to estimate nonspecific binding. All the samples, with four conditions (transfected cells as control, transfected cells with ACh 1 mM, transfected cells with toxic fraction, and transfected cells with toxic fractions and ACh), were incubated for 1h under agitation, at 4 °C. Finally, a final concentration of 5 nM I^125^α-Bgtx was added to the samples. They were well-mixed and incubated for another 1 h at 4 °C. Then, the samples were diluted with 5 mL of phosphate-buffered saline buffer, filtered through GC-filter (Wattman, Fisher Scientific, Waltham, MA, USA), saturated with 5% skim milk, rinsed with 5 mL of phosphate-buffered saline buffer and counted per minute, using a Berthold LB 2111 machine.

Obtained data were normalized to ensure comparability across different experimental conditions. Each condition was tested in triplicate, including the receptor alone, with a positive control, and with the toxic fraction. First, the average counts per minute (CPM) measurement for each triplicate was calculated. To determine specific binding, the average CPM value from the positive control was subtracted from each measurement, to account for non-specific binding. The resulting specific binding values were then normalized by dividing each by the mean specific-binding CPM value of the receptor alone, resulting in normalized values between 0 and 1. This normalization process allowed for accurate comparison of receptor–ligand interactions across different experiments.

### 5.8. Statistical Data Analysis

All data are expressed as mean ± standard error of the mean (SEM). The determination of median lethal doses (LD50) was conducted using the Spearman–Karber method. Curve fitting and half-maximal inhibitory concentration (IC50) calculations were carried out utilizing GraphPad Prism software, version 10.2.0. Differences between control groups and experimental groups, exposed to either toxic fractions or nanobodies, were evaluated using one-way analysis of variance (one-way ANOVA nonparametric). Statistical significance was established at a *p*-value less than 0.05.

## Figures and Tables

**Table 1:** LD50 recorded values of Nh crude venom and toxic fractions in mice using the intraperitoneal injection route. The median lethal doses (LD50) for each component were derived from experiments conducted on groups of four mice, in accordance with the Spearman–Käber method. Each experiment adheres to the minimum statistical number required by the European Pharmacopoeia, and follows ethical guidelines set by the Pasteur Institute of Tunisia. LD50 values, presented in µg/mouse and mg/kg, reflect the final outcomes measured 24 h post administration. To ensure precision, each LD50 determination was verified across three separate trials, including doses both above and below the determined LD50.

N Nh	IP Dose (µg/Mouse)	IP Dose (mg/kg)	Injection Volume (µL)	Mouse Weight (g)	Symptoms
Nht crude Venom	6.64	0.33	500	20 ± 2	Paralysis, difficulty moving, weakness, respiratory distress from muscle paralysis and convulsions.
NhtF5	8.75	0.44	500	20 ± 2	Behavioral difficulties: confusion, tremor, respiratory distress, bending of the back correlated to diaphragm muscle contraction.
NhtF6	14.77	0.74	500	20 ± 2	Flaccid paralysis starting from the hindlimbs and reaching the forelimbs.
Nha crude Venom	5.24	0.26	500	20 ± 2	Weakness, difficulty moving, paralysis, spasm in the abdominal region.
NhaF5	5.63	0.28	500	20 ± 2	Weakness, difficulty moving, paralysis, spasm in the abdominal region.
NhaF6	4.90	0.25	500	20 ± 2	Paralysis, spasm in the abdominal region.

**Table 2:** Preliminary In Vivo Neutralization Results for Different Nanobodies Against NhaF6 in BALB/c Mice via Intraperitoneal Injection.

Nb (Alone or Mixture)	Molar Ratio (NhaF6:Nb)	i.p. NhaF6 LD50 Dose/Mouse	nmol of Nb/Mouse	Survivors/Injected Mice
NhtNbE	1:2	3	5.02	0/5
NhtNbC05	1:2	3	5.02	1/5
NhtNbCl23	1:2	3	5.02	1/5
NhtNbE	1:4	3	10.04	1/5
NhtNbC05	1:4	3	10.04	1/5
NhaNbCl23	1:4	3	10.04	3/5
NhtNbF09	1:4	3	10.04	1/5
NhtNbE NhtNbC05 NhaNbCl23	1:4	3	10.04/Nb	1/5

**Table 3:** In vivo neutralization of NhtF5 by nanobodies via intraperitoneal injection. In vivo neutralization experiments where NhtF5, at a dose of 2LD50 (0.88 mg/kg), was pre-incubated with varying molar ratios of nanobodies (Nbs) before intraperitoneal (i.p.) injection into Swiss mice. The mixture, comprising five Nbs (three targeting Nht (E, F09 and C05) and two targeting Nha (Cl08 and Cl23)), was found to completely neutralize NhtF5 (100%NC). The mixture of five nanobodies at a four-fold molar excess relative to the toxin resulted in a full neutralization in all tested mice (4/4) as highlighted in bold.

Nb (Alone or Mixture)	Molar Ratio (NhtF5:Nb)	i.p. NhtF5 LD50 Dose/Mouse	nmol of Nb/Mouse	Survivors/Injected Mice
NhtNbE	1:4	2	10.04	0/4
NhaNbCl08	1:4	2	10.04	1/4
NhaNbCl23	1:4	2	10.04	2/4
NhtNbE, NhtNbF09, NhtNbC05	1:4	2	10.04/Nb	1/4
NhtNbE, NhtNbF09, NhtNbC05, NhaNbCl08, NhaNbCl23	1:1	2	2.51/Nb	1/4
NhtNbE, NhtNbF09, NhtNbC05, NhaNbCl08, NhaNbCl23	1:2	2	5.02/Nb	2/4
**NhtNbE,** **NhtNbF09, NhtNbC05, NhaNbCl08, NhaNbCl23**	**1:4**	**2**	**10.04/Nb**	**4/4**

## Data Availability

The raw data supporting the conclusions of this article will be made available by the authors on request.

## Supplementary Materials

The following supporting information can be downloaded at: https://www.mdpi.com/article/10.3390/toxins16090393/s1, Figure S1: Immune Response Curve of the Dromedary Following Immunization with Cobra Venom Fractions, Figure S2: Identification of the 90 Positive Rescued Clones Specific to NhtF5 and NhtF6 (a) and 2 clones specific to NhaF6 (b), Figure S3: SDS-PAGE analysis of purified nanobodies expressed in prokaryotic systems; Figure S4: Biochemical analysis of the purified monovalent nanobodies, produced in HEK 293 eukaryotic cells, using SDS-PAGE; Figure S5: Gel filtration analysis of purified nanobodies; Table S1: LD50 recorded values of Nh crude venom and toxic fractions in mouse using Intraperitoneal Injection route; Table S2: Properties of Nanobodies Used in Neutralization Studies. Analyzed Using Expasy (V3.0) (https://web.expasy.org/compute_pi/ accessed on 19 July 2024); Table S3: List of the primers used for the construction of the VHH libraries. (a) Primers used for PCR1. (b) Primers used for PCR2 (restriction enzyme sites are underlined in the primer sequences above).
