# Supplementary material for: Neutralizing Nanobodies against Venoms from Naja haje Species Captured in North Africa
Source: Toxins (Basel). 2024 Sep 14;16(9):393. doi: 10.3390/toxins16090393 (PMC11435604; doi:10.3390/toxins16090393)
Supplement: Supplementary file 1 [file toxins-16-00393-s001.zip › toxins-3169961-supplementary.pdf]

Supplementary data

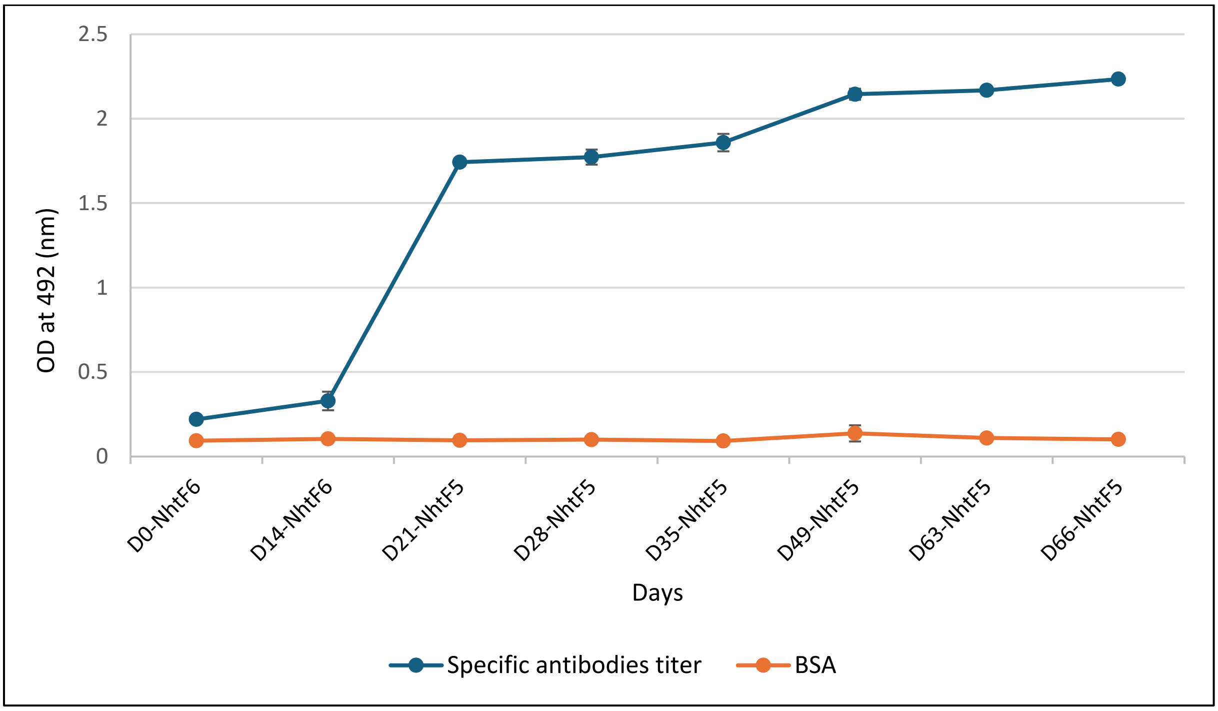

Figure S1: Immune Response Curve of the Dromedary Following Immunization with Cobra Venom Fractions

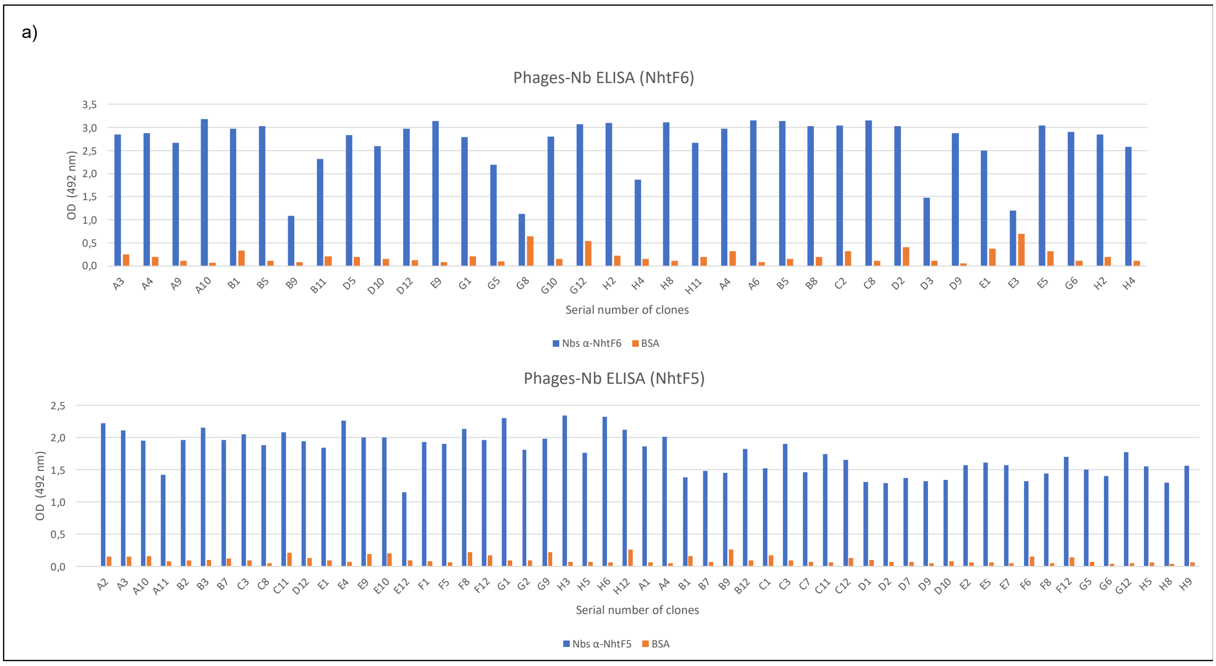

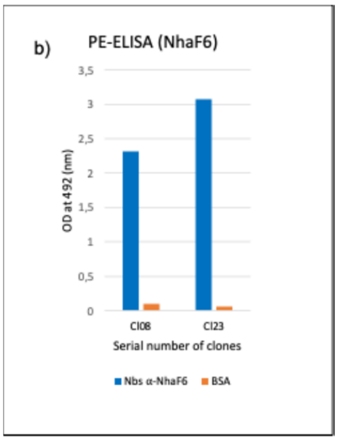

Figure S2: Identification of the 90 Positive Rescued Clones. (a) Rescued Clones Specific to NhtF5 and NhtF6 (b) Rescued clones specific to NhaF6.

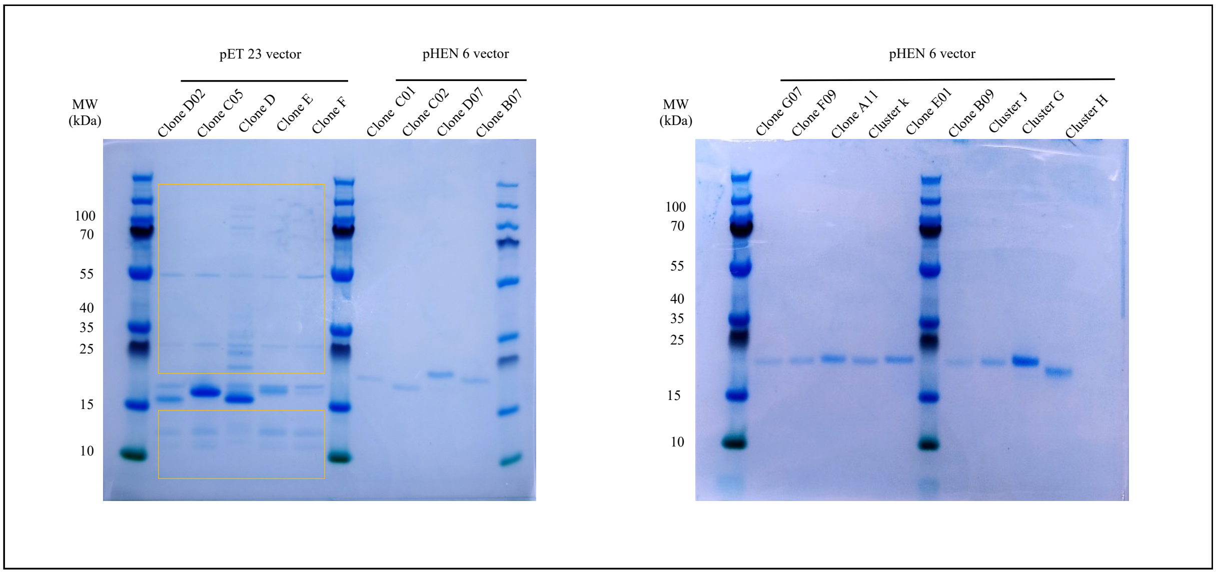

Figure S3: SDS-PAGE analysis of purified nanobodies expressed in prokaryotic systems. All produced nanobodies were analyzed by sodium dodecyl sulfate-polyacrylamide gel electrophoresis (SDS-PAGE). Samples were heated to 95°C in a 4x LDS sample buffer containing β-mercaptoethanol for 10 minutes and then analyzed on a 12% SDS-PAGE gel. Biochemical analysis of the selected monovalent nanobodies using SDS-PAGE is shown: lanes 2-6 represent the eluted fractions containing the purified NhtF5/NhtF6 nanobodies produced in pET23a (cytoplasmic expression, and the remaining lanes represent the eluted fractions containing the purified NhtF5/NhtF6 nanobodies produced in pHEN6 (periplasmic expression. The nanobodies were successfully produced and expressed at the expected size. The bands above and below the nanobodies (highlighted in yellow) are histidine-rich chaperone proteins, which appear exclusively after nanobody expression in pET23a). These nanobodies, which contained contaminants, were further purified using gel filtration (Cytiva-ÄKTA). The nanobodies were successfully produced and expressed at the expected size.

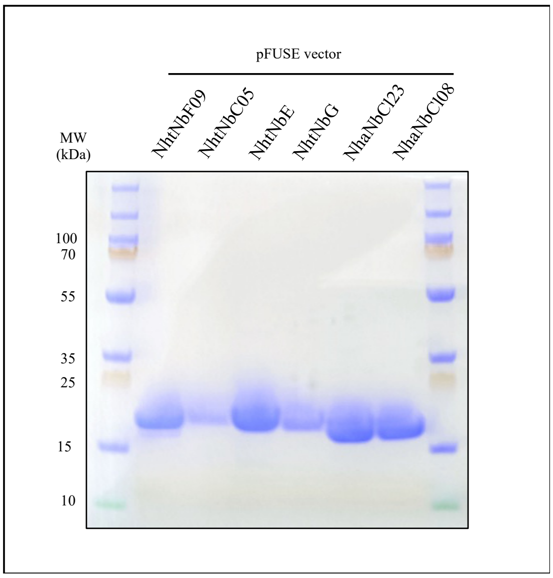

Figure S4: Biochemical analysis of the purified monovalent nanobodies, produced in HEK 293 eukaryotic cells, using SDS-PAGE.

Five nanobodies with NhaF6-neutralizing capacities, including a negative control nanobody, were subcloned into a eukaryotic system. Nanobodies were purified using cobalt HisPur beads on an Econo-pack column, followed by gel filtration (Cytiva-ÄKTA). Purity was assessed by SDS-PAGE and visualized with Coomassie blue staining. The nanobodies presented intense bands with different molecular weights (around 15 kDa) due to structural variations.

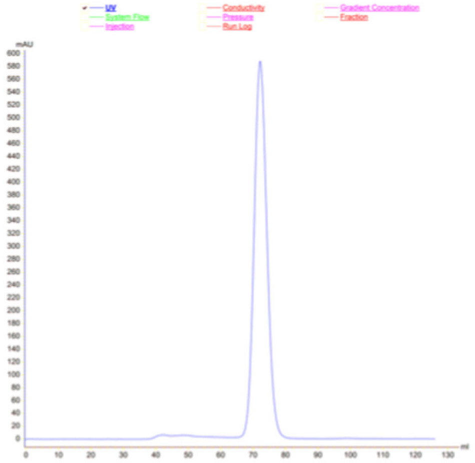
UV
System Flow
Injection
Conductivity
Pressure
Run Log
Gradient Concentration
Fraction
mAU
ml

(a)

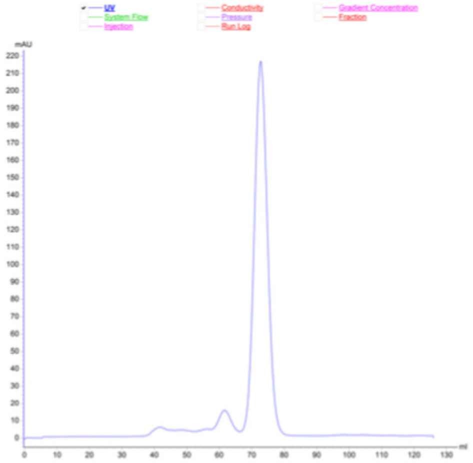
UV
System Flow
Injection
Conductivity
Pressure
Run Log
Gradient Concentration
Fraction
mAU
ml

(b)

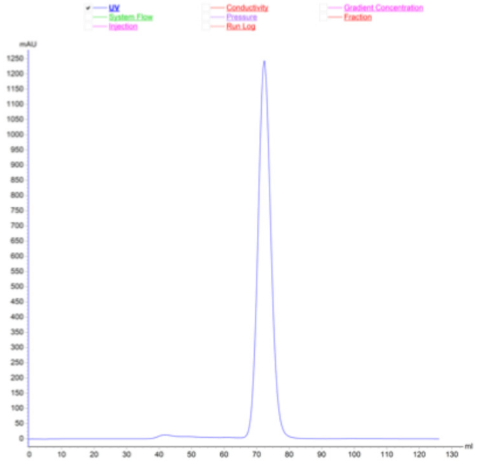
UV
System Flow
Injection
Conductivity
Pressure
Run Log
Gradient Concentration
Fraction
mAU
ml

(c)

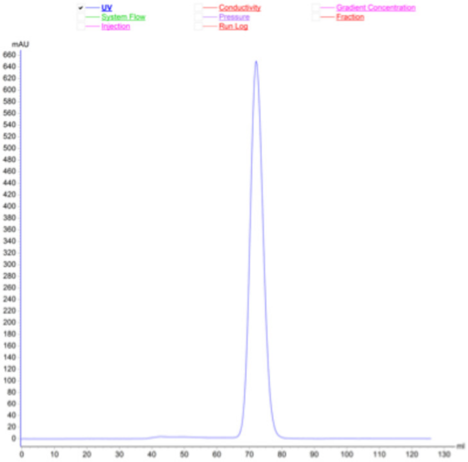
UV
System Flow
Injection
Conductivity
Pressure
Run Log
Gradient Concentration
Fraction
mAU
ml

(d)

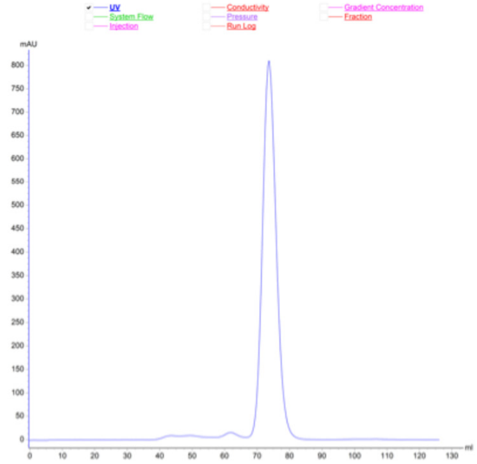
UV
System Flow
Injection
Conductivity
Pressure
Run Log
Gradient Concentration
Fraction
mAU
ml

(e)

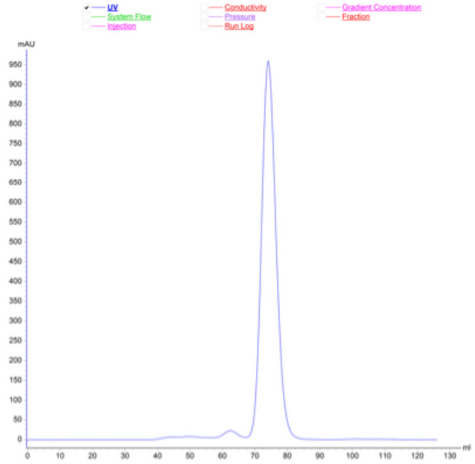
UV
System Flow
Injection
Conductivity
Pressure
Run Log
Gradient Concentration
Fraction
mAU
ml

(f)

Figure S5: Gel filtration analysis of purified nanobodies. The chromatograms are identified as follows: (a) Nanobody NhtNbG, (b) Nanobody NhtNbC05, (c) Nanobody NhtNbF09, (d) Nanobody NhtNbE, (e) Nanobody NhaNbCl08, and (f) Nanobody NhaNbCl23.

Nh	IP dose (µg)/mouse	Injection Volume (µl)	Mouse weight (g)	Survivors/injected mouse	Symptoms
Nht crude Venom	7.26	500	20 g ± 2	0/4	**Paralysis, difficulty moving, weakness, respiratory distress from muscle paralysis and convulsions**
	7.03	500	20 g ± 2	1/4	
	6.81	500	20 g ± 2	1/4	
	**6.6**	**500**	**20 g ± 2**	**2/4**	
	6.4	500	20 g ± 2	3/4	
	6.31	500	20 g ± 2	4/4	
**NhtF5**	9.43	500	20 g ± 2	0/4	**Behavioral problems: confusing, tremor, muscle spasms**
	9.21	500	20 g ± 2	0/4	
	9.0	500	20 g ± 2	4/4	
	**8.8**	**500**	**20 g ± 2**	**2/4**	
	8.59	500	20 g ± 2	3/4	
	8.4	500	20 g ± 2	3/4	

	8.21	500	20 g ± 2	4/4	
**NhtF6**	15.4	500	20 g ± 2	0/4	**Paralyzed starting from the hindlimbs reaching the forelimbs**
	15.2	500	20 g ± 2	1/4	
	**15**	**500**	**20 g ± 2**	**2/4**	
	14.8	500	20 g ± 2	3/4	
	14.6	500	20 g ± 2	3/4	
	14.4	500	20 g ± 2	3/4	
	14.2	500	20 g ± 2	3/4	
	14	500	20 g ± 2	4/4	
***Nha* crude Venom**	10.36	500	20 g ± 2	4/4	
	8.28	500	20 g ± 2	4/4	
	6.62	500	20 g ± 2	3/4	
	**5.29**	**500**	**20 g ± 2**	**2/4**	
	4.24	500	20 g ± 2	1/4	
	3.39	500	20 g ± 2	0/4	
**NhaF5**	10.6	500	20 g ± 2	4/4	
	8.48	500	20 g ± 2	4/4	

	6.784	500	20 g ± 2	3/4
	**5.43**	**500**	**20 g ± 2**	**2/4**
	4.3	500	20 g ± 2	0/4
**NhaF6**	8.48	500	20 g ± 2	4/4
	6.784	500	20 g ± 2	3/4
	**5.43**	**500**	**20 g ± 2**	**2/4**
	4.3	500	20 g ± 2	1/4
	3.44	500	20 g ± 2	0/4

Table S1: LD50 recorded values of Nh crude venom and toxic fractions in mouse using Intraperitoneal Injection route using the Spearman-Kärber method

	NhtNbC05	NhtNbF09	NhtNbE	NhaNbCl08	NhaNbCl23
Molecular Weight (Da)	16671.43	17040.84	17015.58	16047.72	16017.70
Isoelectric Point (pI)	6.28	5.81	5.40	6.03	6.03
CDR3 Length	20	23	20	17	17

	NhtNbC05	NhtNbF09	NhtNbE	NhaNbCl08	NhaNbCl23
Molecular Weight (Da)	13992.60	14303.98	14349.80	13321.80	13136.60
Isoelectric Point (pI)	7.71	6.17	5.04	6.54	6.38
CDR3 Length	20	23	20	17	17

Table S2: Properties of Nanobodies Used in Neutralization Studies. Analyzed Using Expasy (https://web.expasy.org/compute_pi/).

a)

**Primer**	**Sequence(5′–3′)**
VHBACK A6	5′ GATGTGCAGCTGCAGGCGTCTGGRGGAGG 3′

CH2FORTA4	5′ CGCCATCAAGGTACCAGTTGA 3′
ALPAGA (peptide leader)	5′- GGTGGTCCTGGCTGC -3′
ALPAGA (CH2)	5′- ATGGAGAGGACGTCCTTGGGT-3′
CALL001 (peptide leader)	5′- GTCCTGGCTGCTCTCTACAAGG-3′
CALL002 (CH2)	5′- GGTACGTGCTGTTGAACTGTTCC -3′
Bq-lead-short2-F	5′ G GTG GTC CTG GCT GCN CT 3′
Bq-CH2-R	5′ ATG GAG AGG ACG TCC TTG GGT 3′
Bq-lead-long-F	5′ GTCCTGGCTGCTCTWYTACARGG 3′
Bq-CH2-call2-R	5′ GGTACGTGCTGTTGAACTGTTCC 3′

b)

**Primer**	**Sequence(5′–3′)**
VHBACKA4	5′ CATGCCATGACTCGCGGCCCAGCCGGCCATGGCCGAKGTSCAGCT 3′
VHFOR36	5′ GGACTAGTTGCGGCCGCTGAGGAGACGGTGACCTG 3′
Bq-FR1-long-F	5′ GTCATTGGCCCAGCCGGCCATGGCTCAGKTGCAGCTCGTGGAGTCNGG 3′
Bq-Hinge-short-R	5′ GACATTGCGGCCGCGCTGGGGTCTTCGCTGTGGTG 3′
Bq-FR1-long-F	5′ GTCATTGGCCCAGCCGGCCATGGCTCAGKTGCAGCTCGTGGAGTCNGG 3′
Bq-Hinge-long-R	5′ GACATTGCGGCCGCTGGTTGTGGTTTTGGTGTCTTGGG 3′

Table S3: List of the primers used for the construction of the VHH libraries. (a) Primers used for PCR1. (b) Primers used for PCR2 (restriction enzyme sites are underlined in the primer sequences above).
